# The Role of Dietary Antioxidants, Food Supplements and Functional Foods for Energy Enhancement in Healthcare Professionals

**DOI:** 10.3390/antiox13121508

**Published:** 2024-12-10

**Authors:** Theodora Kalogerakou, Maria Antoniadou

**Affiliations:** 1 Department of Dentistry, School of Health Sciences, National and Kapodistrian University of Athens, 11527 Athens, Greece; 2 Executive Mastering Program in Systemic Management (CSAP), University of Piraeus, 18534 Piraeus, Greece

**Keywords:** oxidative stress, dietary antioxidants, food supplements, functional foods, healthcare professionals

## Abstract

Healthcare professionals frequently experience significant work overload, which often leads to substantial physical and psychological stress. This stress is closely linked to increased oxidative stress and a corresponding decline in energy levels. This scoping review investigates the potential impact of dietary antioxidants and food supplements in conjunction with diet in controlling these negative effects. Through an analysis of the biochemical pathways involved in oxidative stress and energy metabolism, the paper emphasizes the effectiveness of targeted dietary interventions. Key dietary antioxidants, such as vitamins C and E, polyphenols, and carotenoids, are evaluated for their ability to counteract oxidative stress and enhance energy levels. Additionally, the review assesses various food supplements, including omega-3 fatty acids, coenzyme Q10, and ginseng, and their mechanisms of action in energy enhancement. Practical guidelines for incorporating energy-boost dietary strategies into the routine of healthcare professionals are provided, emphasizing the importance of dietary modifications in reducing oxidative stress and improving overall well-being and performance in high-stress healthcare environments. The review concludes by suggesting directions for future research to validate these findings and to explore new dietary interventions that may further support healthcare professionals under work overload.

## 1. Introduction

Healthcare professionals, including doctors, nurses, and allied health workers, operate in environments marked by high stress, long hours, and the constant need for critical decision making (2020) [1]. They operate within healthcare organizations in a so-called VUCA framework—Volatility, Uncertainty, Complexity, and Ambiguity [2]. The volatility of healthcare arises from constantly evolving patient needs, unpredictable workloads, and rapid advancements in medical technology, all of which place additional stress on professionals [3]. Uncertainty compounds this stress, as healthcare workers frequently face incomplete information and must make critical decisions under pressure [4]. The complexity and ambiguity of healthcare settings further intensify this burden, as professionals work through intricate systems of care, interpret complex medical data, and adapt to shifting protocols while striving to maintain high-quality patient outcomes [5].

Such demanding conditions often result in significant physical and psychological strain, which has been linked to increased oxidative stress in a cellular base, particularly through the production of reactive oxygen species (ROS) [6]. These ROS can overwhelm the body’s antioxidant defenses, leading to cellular damage and energy depletion [7]. At a cellular level, oxidative stress occurs when there is an imbalance between the production of free radicals and the body’s ability to counteract their harmful effects through antioxidants [8,9]. Free radicals such as ROS are by-products of normal metabolic processes [10], but under stressful conditions—such as those experienced by healthcare professionals—their production can increase significantly, disrupting the balance between free radicals and antioxidants and leading to cellular damage and energy depletion [11,12]. This depletion of energy reserves diminishes healthcare professionals’ ability to manage stressful situations, creating a vicious cycle of stress and energy loss [13]. The oxidative stress experienced in healthcare environments is further enhanced by prolonged exposure to stressors, which accelerates oxidative damage [9], speeds up the aging process [14], and contributes to chronic health issues such as burnout, fatigue, and cognitive decline [15].

Healthcare professionals are particularly vulnerable to these conditions and are driven quite often to burnout [16]. Burnout, characterized by emotional exhaustion, depersonalization, and a reduced sense of personal accomplishment, is strongly connected to oxidative stress [16,17]. Chronic workplace stress not only increases oxidative damage but also impairs mitochondrial function, leading to decreased ATP synthesis and chronic fatigue [13,18]. The resulting energy depletion corresponds to both physical and psychological distress, further undermining healthcare professionals’ ability to manage demanding workloads [19].

Furthermore, emerging research emphasizes that targeted antioxidant supplements, particularly those containing key compounds such as coenzyme Q10 and omega-3 fatty acids, are critical in reducing oxidative stress and supporting energy production [20]. Coenzyme Q10, for example, has been shown to enhance mitochondrial function, protecting against oxidative damage and improving ATP synthesis [21]. Similarly, omega-3 fatty acids have demonstrated significant anti-inflammatory and antioxidant properties, which are essential for modulating oxidative stress and enhancing energy levels in high-stress environments [22,23]. Also, there are functional foods such as probiotic yogurt, fortified cereals, oats, and dark chocolate that contain biologically active compounds such as vitamins, minerals, fiber, or probiotics, which are believed to enhance health, prevent disease, or improve physical and mental well-being [24]. Unlike dietary supplements and antioxidants, which are concentrated forms of nutrients or active compounds, functional foods are consumed as part of a regular diet. All antioxidant supplements and other functional foods offer great potential for healthcare professionals, who require sustained energy and cognitive function to cope with their demanding work environments [20,21,24].

While there is extensive research on oxidative stress in stressful work environments, fewer studies address the targeted use of dietary antioxidants specifically in healthcare workers [25]. The purpose of this paper is thus to examine the role of dietary antioxidants and food supplements in reducing oxidative stress and enhancing energy depletion among healthcare professionals. Specifically, this paper aims to identify antioxidants and supplements that are effective in this context, study their biochemical mechanisms, and propose evidence-based dosage recommendations for this population group. Through these objectives, the study seeks to offer practical, science-based dietary interventions that can enhance healthcare professionals’ well-being, energy sustainability, and performance.

## 2. Methodology

Our search strategy for this narrative review aimed to capture a broad spectrum of literature related to dietary intervention and the use of antioxidants and supplements for stress and energy management in healthcare professionals. We followed the methodological framework proposed by Arksey and O’Malley (2005) [26], as refined by Levac, Colquhoun, and O’Brien (2010) [27]. To identify relevant studies, a comprehensive search strategy was employed, targeting electronic databases such as PubMed, Scopus, and Web of Science. The search focused on articles published from 2000 to the present, using a combination of keywords such as “oxidative stress”, “dietary antioxidants”, “food supplements”, “functional foods”, “healthcare professionals”, “burnout”, “energy depletion”. Boolean operators (AND/OR) were applied to refine the search, and filters such as peer-reviewed articles in English were used to ensure the quality of the studies. More specifically, the inclusion criteria consisted of articles, systematic reviews, randomized controlled trials (RCTs), cohort studies, case–control studies, and qualitative research investigating dietary interventions to reduce oxidative stress and enhance energy levels. Studies focused specifically on healthcare professionals such as doctors, dentists, nurses, and allied health workers were included [28]. Additionally, studies involving technological tools for stress monitoring and nutritional optimization were considered. Exclusion criteria included studies that focus on non-healthcare populations, animal studies, and those that do not assess dietary interventions related to oxidative stress.

Furthermore, the study involved two independent reviewers who first screened titles and abstracts to identify studies meeting the inclusion criteria. Full-text reviews of potentially eligible studies were conducted to confirm their suitability. The data extraction was carried out using a standardized data extraction form designed to capture key information from the included studies [29]. The extracted data included study characteristics such as authorship, year of publication, country of study, and study design. Population details, including healthcare professionals’ roles, demographics, and work environment, were recorded. Intervention details were focused on the types of dietary antioxidants, supplements, functional foods, and technological tools used, while outcomes related to oxidative stress reduction, energy enhancement, and overall well-being were documented, too. Finally, the data were synthesized descriptively and organized based on the type of dietary intervention, the observed outcomes (such as reductions in oxidative stress or improvements in energy levels), and the effectiveness of technological tools for monitoring and optimizing these interventions. The reporting of our findings adheres to the PRISMA-ScR guidelines (Preferred Reporting Items for Systematic reviews and Meta-Analyses extension for Scoping Reviews) (https://www.prisma-statement.org/prisma-2020-flow-diagram, accessed on 20 September 2024) [30]. Finally, ethical approval was not required because this review involves only publicly available data.

## 3. Results

### 3.1. Oxidative Stress in Healthcare Professionals and Mechanisms

As is already reported, oxidative stress occurs when there is an imbalance between the production of ROS and the body’s ability to neutralize these reactive molecules through antioxidants [31,32]. ROS are highly reactive molecules containing oxygen, including free radicals such as superoxide (O_2_^−^) and hydroxyl (OH^−^) radicals, as well as non-radical species like hydrogen peroxide (H_2_O_2_) [33,34]. These molecules are by-products of normal cellular metabolism, particularly those of the ATP generation process in the mitochondria [33,35]. Under normal conditions, ROS play a role in cell signaling and homeostasis, but when their levels become excessive, they can cause significant cellular damage [36].

Mitochondria, often referred to as the “powerhouses of the cell”, are both the primary source and target of ROS [37,38]. During the process of oxidative phosphorylation, electrons leak from the electron transport chain and react with oxygen to form superoxide [39]. Under stressful conditions, such as those experienced by healthcare professionals, the rate of mitochondrial ROS production increases, overwhelming the body’s antioxidant defenses [40,41]. This leads to mitochondrial dysfunction, further exacerbating ROS production and contributing to energy depletion [42]. In this sense, antioxidants are crucial for countering the effects of ROS. Endogenous antioxidants, such as superoxide dismutase (SOD), catalase, and glutathione peroxidase, work to neutralize ROS and prevent oxidative damage [33]. However, during periods of elevated oxidative stress, such as chronic exposure to high workloads and emotional stress in healthcare environments, the body’s endogenous antioxidants can become overwhelmed, necessitating additional support from exogenous antioxidants obtained through diet or supplements [43].

### 3.2. Impact of Oxidative Stress on Healthcare Professionals

Oxidative stress has a great impact on the health of individuals and, more specifically, on the health of healthcare professionals [16]. One of the most significant effects of oxidative stress on healthcare workers is its detrimental impact on cognitive function [11]. Studies have shown that chronic exposure to oxidative stress can lead to neuroinflammation, which in turn impairs cognitive abilities such as memory, attention, and decision making [16]. These cognitive impairments are particularly concerning in healthcare settings, where professionals are required to make quick and accurate decisions, often under pressure [40]. For example, high levels of oxidative stress can reduce the efficiency of the prefrontal cortex, a brain region critical for executive functions and decision making [44].

Additionally, oxidative stress has been closely linked to mood disorders such as anxiety and depression [40]. Healthcare professionals frequently experience burnout, a condition marked by emotional exhaustion, depersonalization, and a reduced sense of personal accomplishment, which is exacerbated by oxidative stress [45]. ROS can disrupt the balance of neurotransmitters, such as serotonin and dopamine, that regulate mood, leading to increased susceptibility to mood disorders [46]. For instance, studies have found that healthcare workers with higher oxidative stress levels report more symptoms of anxiety and depression compared to their peers with lower oxidative stress [43,47]. Moreover, chronic oxidative stress can accelerate the aging process and contribute to the development of neurodegenerative diseases such as Alzheimer’s and Parkinson’s disease, both of which are characterized by cognitive decline [48]. In healthcare professionals, who are already at risk due to the high-stress nature of their work, this connection is particularly concerning. Long-term exposure to oxidative stress can lead to the accumulation of oxidative damage in the brain, which accelerates neuronal degeneration and impairs cognitive function over time [49].

Cognitive impairment can elevate stress levels, leading to a vicious cycle where increased stress further elevates oxidative stress, which in turn impairs cognitive abilities even more [39]. This cycle can lead to chronic fatigue, burnout, and, ultimately, reduced job performance [16]. As such, healthcare workers who experience cognitive decline and mood disorders are less able to cope with the demands of their jobs, which can compromise patient care [4].

In addition to cognitive and psychological effects, oxidative stress also has significant implications for the long-term physical health of healthcare professionals. Chronic oxidative stress is a known risk factor for cardiovascular diseases, which are prevalent among individuals working in high-stress professions [50]. ROS contributes to the development of atherosclerosis by oxidizing low-density lipoprotein (LDL) cholesterol, which leads to plaque formation in arteries [51]. This process increases the risk of heart attacks and strokes, which are major health concerns for healthcare professionals, who often neglect their health while prioritizing patient care [16]. Oxidative stress has also been linked to metabolic disorders such as diabetes and obesity, both of which are more prevalent among healthcare workers due to irregular working hours, poor diet, and lack of physical activity [52]. Also, ROS can disrupt insulin signaling pathways, leading to insulin resistance, which is a precursor to type 2 diabetes [53]. Furthermore, oxidative stress can promote the accumulation of adipose tissue, contributing to obesity, which further increases the risk of cardiovascular diseases [43].

The impact of oxidative stress on immune function is another critical concern for healthcare workers, who are frequently exposed to infectious agents [54]. Chronic oxidative stress impairs the immune response by damaging immune cells and reducing their ability to fight infections [55]. Healthcare professionals with weakened immune systems are more susceptible to infections, which can lead to increased absenteeism and compromised patient care [16], while impaired immune function can also lead to the development of autoimmune diseases [11]. Further, oxidative stress contributes to the development of cancer, another long-term health risk for healthcare professionals [56]. ROS can cause mutations in DNA, which can lead to the uncontrolled growth of cells, leading to cancer [56]. Healthcare workers who are exposed to radiation, chemicals, and other carcinogenic agents in the workplace are particularly vulnerable to the combined effects of oxidative stress and environmental toxins, too [57]. Studies have shown that healthcare professionals in high-stress environments are at an increased risk of developing cancers such as breast, lung, and colorectal cancer [56]. Overall, the cumulative impact of this kind of stress on cognitive function, mood, and long-term health makes it a significant occupational hazard for healthcare professionals [57].

**Figure 1:**
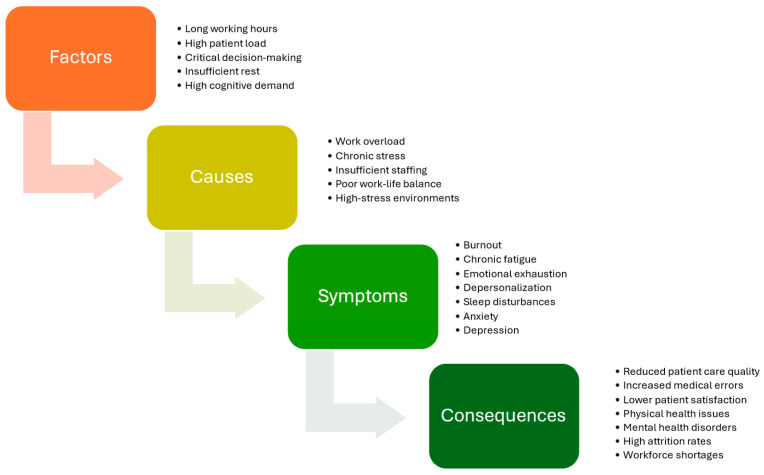
Factors, causes, symptoms, and consequences of work-related stress in healthcare professionals.

In conclusion, long working hours, high patient load, critical decision making, insufficient rest, and high cognitive demand are key factors that contribute to work overload, chronic stress, insufficient staffing, poor work–life balance, and high-stress environments [58]. These causes result in symptoms such as burnout, chronic fatigue, emotional exhaustion, depersonalization, sleep disturbances, anxiety, and depression. The consequences of these symptoms are severe, leading to reduced patient care quality, increased medical errors, lower patient satisfaction, physical and mental health issues among healthcare professionals, and workforce shortages [59,60]. This highlights the critical need for addressing work-related stress in healthcare settings to protect both healthcare workers and the quality of patient care. In Figure 1, the relationship between factors, causes, symptoms, and consequences of work-related stress in healthcare professionals is presented.

### 3.3. Mechanisms and Benefits of Dietary Antioxidants

One of the most recommended methods for managing oxidative stress in healthcare professionals is dietary modification. Consuming a diet rich in antioxidants can help neutralize the harmful effects of ROS and reduce oxidative damage [7]. Antioxidant supplements are essential compounds that help protect the body from oxidative stress by neutralizing ROS and preventing cellular damage [7,8,9]. These supplements work by donating electrons to ROS, stabilizing them and preventing them from damaging important cellular components such as DNA, proteins, and lipids [51,61]. So, antioxidants play a crucial role in maintaining health and preventing chronic diseases by reducing oxidative stress [62,63].

Μοre specifically, vitamin C, also known as ascorbic acid, is one of the most potent water-soluble antioxidants and plays a key role in scavenging free radicals [43]. It acts as a first line of defense by directly neutralizing ROS in the aqueous parts of the body, including blood and intracellular fluid [64]. Additionally, vitamin C regenerates other antioxidants, such as vitamin E, back to their active forms, further enhancing its protective role [43]. A diet rich in citrus fruits, strawberries, and bell peppers provides high levels of vitamin C, helping to combat oxidative stress [65]. Further, vitamin E is a lipid-soluble antioxidant that primarily protects cell membranes from lipid peroxidation, a process by which ROS damage the lipids in cell membranes, leading to cell death [65]. As a key component in protecting polyunsaturated fatty acids in cell membranes, vitamin E plays a critical role in maintaining membrane integrity and cellular function [43,55]. Sources of vitamin E include nuts, seeds, and vegetable oils, making them important components of a diet designed to reduce oxidative stress [43,57].

In addition, polyphenol-based antioxidant supplements, such as flavonoids and phenolic acids, are a powerful class of compounds that have been shown to upregulate the body’s endogenous antioxidant defenses [66]. These supplements enhance the activity of antioxidant enzymes like superoxide dismutase (SOD) and catalase, further improving the body’s ability to neutralize ROS [51]. Clinical studies have shown that polyphenol-rich supplements can reduce oxidative stress and inflammation, supporting the health and energy levels of healthcare professionals working in high-stress environments [62]. Also, carotenoid-based antioxidant supplements, including beta-carotene, lycopene, lutein, and zeaxanthin, play a critical role in protecting against oxidative damage [63,67]. These supplements are particularly effective at neutralizing singlet oxygen, a highly reactive form of ROS, and are essential for protecting cellular functions, especially in high-stress environments faced by healthcare professionals [68]. Carotenoid supplementation has been linked to improved visual health and a reduction in oxidative stress-related conditions, such as age-related cellular damage [52]. Finally, studies have demonstrated that antioxidant supplements play a crucial role in modulating signaling pathways involved in oxidative stress and inflammation [69,70]. For instance, polyphenol supplements have been shown to activate the nuclear factor erythroid 2–related factor 2 (Nrf2) pathway, enhancing the body’s antioxidant defenses and supporting healthcare professionals by reducing oxidative stress [71,72]. This dual role in neutralizing ROS and regulating oxidative stress pathways makes targeted antioxidant supplementation essential for managing inflammation and preventing chronic disease in healthcare professionals [73].

#### 3.3.1. Classification and Sources of Antioxidants

Antioxidant supplements can be broadly classified into two categories, endogenous and exogenous, based on their source of origin [74]. Endogenous antioxidants are naturally produced within the body and play a crucial role in neutralizing ROS generated during normal metabolic processes [75]. These include key enzymes such as superoxide dismutase (SOD), catalase, and glutathione peroxidase, which are the body’s first line of defense against oxidative stress [63]. For instance, SOD converts superoxide radicals into hydrogen peroxide, which is then broken down by catalase into water and oxygen, preventing oxidative damage to cells and tissues [76].

Endogenous antioxidant supplements play a crucial role in the body’s defense against oxidative stress, acting within cells to neutralize ROS [77]. One of the most important endogenous antioxidants is glutathione, often referred to as the “master antioxidant” [63]. Glutathione is essential for detoxifying cells, recycling other antioxidants like vitamin C and E, and maintaining immune function [78]. It neutralizes free radicals and is critical for the detoxification of harmful substances, particularly in high-stress environments like the healthcare setting [62]. SOD is another key endogenous antioxidant that converts superoxide radicals into hydrogen peroxide, which is then broken down by catalase, preventing oxidative damage to cells [79]. SOD, catalase, and glutathione peroxidase together form the body’s first line of defense against oxidative stress in healthcare professionals experiencing prolonged stress [80].

Exogenous antioxidant supplements, on the other hand, are obtained from external sources and are crucial for supporting the body’s natural defenses when endogenous mechanisms are overwhelmed by excessive ROS production [81]. Supplements containing compounds such as polyphenols, carotenoids, and flavonoids work synergistically with endogenous antioxidants to reduce oxidative stress [51,82]. For example, polyphenol-based supplements have shown strong antioxidant properties, supporting cardiovascular health and reducing inflammation [71]. Also, carotenoid supplements, such as beta-carotene and lycopene, are essential for maintaining cellular health, particularly under the high demands placed on healthcare professionals [63,83].

Both endogenous and exogenous antioxidant supplements are necessary for maintaining the body’s redox balance and preventing oxidative damage in high-stress professions like healthcare [69,84]. Together, these supplements support overall health by reducing the risks associated with chronic oxidative stress and maintaining energy levels [85].

#### 3.3.2. The Role of Antioxidants in Energy Enhancement

Antioxidant supplements such as vitamin E and coenzyme Q10 help protect mitochondria from oxidative damage, thereby preserving their ability to produce energy, which is essential for sustaining high performance in demanding healthcare environments [86]. Coenzyme Q10, in particular, plays a key role in mitochondrial energy production by supporting ATP synthesis and reducing oxidative stress in healthcare professionals working under high-stress conditions [87]. Additionally, polyphenol-based supplements have been reported to support mitochondrial function by enhancing the activity of mitochondrial enzymes involved in ATP production [88]. Polyphenols, such as those found in green tea extract supplements, have been shown to improve mitochondrial efficiency and reduce oxidative damage to mitochondrial membranes, further supporting energy production and reducing fatigue [69,71].

Moreover, research has shown that antioxidants often work synergistically, meaning their combined effect is greater than the sum of their actions [43]. For example, vitamin C regenerates oxidized vitamin E, restoring its antioxidant function and allowing it to continue protecting cell membranes from oxidative damage [89]. Similarly, polyphenol-based supplements can enhance the activity of endogenous antioxidants like glutathione peroxidase, creating a stronger defense against ROS [51,90]. These antioxidant supplements work synergistically to neutralize ROS, protecting against oxidative damage in healthcare professionals exposed to high levels of stress [71]. In conclusion, the combination of supplements, such as vitamins C and E, polyphenols, and carotenoids, allows healthcare professionals to gain comprehensive protection against the harmful effects of oxidative stress [91,92].

#### 3.3.3. Mediterranean Diet and Practical Dietary Recommendations with Antioxidants for Healthcare Professionals

The Mediterranean diet has been shown to offer significant health benefits, particularly for healthcare professionals who face high levels of stress and long working hours. Its high content of omega-3 fatty acids from olive oil and fatty fish helps reduce inflammation and oxidative stress, common issues in healthcare settings [93]. This diet’s antioxidants, such as vitamin C and polyphenols from fruits, vegetables, and nuts, neutralize free radicals, lowering oxidative stress and inflammation, which supports both physical and cognitive health [94]. Omega-3s and polyphenols have been linked to improved cognitive function and mental resilience, both of which are critical for healthcare professionals. These nutrients are essential for brain health, enhancing memory, and reducing the risk of depression, which is often exacerbated by stressful work environments [94]. By maintaining mental clarity and energy, omega-3s and polyphenols are ideal for healthcare workers who require sustained focus and vitality during long shifts [94]. Furthermore, this nutrient-rich diet supports cardiovascular health by promoting healthy blood pressure and cholesterol levels, thereby reducing the risk of heart disease, a common issue for healthcare professionals under chronic stress [93]. The combination of heart-healthy fats, fiber, and antioxidants in such a diet helps to sustain cardiovascular function, which is crucial for maintaining overall well-being in high-pressure environments [93].

Apart from the diet, antioxidant supplements play a crucial role in restoring the balance between ROS production and antioxidant defenses, thereby reducing the risk of oxidative damage and its associated health consequences [95]. Healthcare professionals can benefit from targeted supplementation, which includes antioxidants such as vitamins C and E, coenzyme Q10, and polyphenols, to support their ability to manage oxidative stress in demanding environments [51].

In Table 1, we present the natural sources of the most common antioxidants recommended for usage and Mediterranean diet tips for healthcare professionals.

Overall, vitamin C (ascorbic acid) supplementation is recommended at 75–90 mg per day for adults, with smokers needing an additional 35 mg [96]. Also, polyphenol supplements, commonly taken at doses of 200–1000 mg/day, with therapeutic doses reaching up to 1500 mg/day, modulate inflammation, protect DNA, support vascular health, and improve cognitive function in high-stress environments [71]. Carotenoid supplements, such as beta-carotene, lycopene, and lutein, are recommended in specific doses: lutein at 6–15 mg/day and lycopene at 2–4 mg/day, providing support for eye, skin, and immune health as well as cognitive health in older adults [97,98]. Moreover, astaxanthin supplements are typically consumed at 4–12 mg/day, with higher doses of up to 16 mg/day recommended for athletes to enhance performance [62]. Also, lipoic acid supplements are recommended at 300–600 mg/day, with up to 1200 mg/day for individuals with diabetes [71]. Lipoic acid regenerates other antioxidants, supports energy metabolism, and is beneficial for cognitive and glucose metabolism [69].

Further, glutathione is typically supplemented at 250–500 mg/day and acts as a master antioxidant, aiding detoxification and boosting immune function, especially in elderly populations [99]. Further, N-acetyl cysteine (NAC), a derivative of cysteine, is taken as a supplement in doses of 600–1200 mg/day, with higher doses used for lung health. NAC serves as a precursor to glutathione and is useful in managing neurodegenerative disorders [51]. Also, silymarin, derived from milk thistle, supports liver detoxification, reduces lipid peroxidation, and enhances antioxidant enzyme activity, with recommended doses of 200–400 mg/day, though pregnant women should avoid it [51]. In addition, curcumin, found in turmeric, is taken in doses of 500–2000 mg/day for its anti-inflammatory and antioxidant properties, supporting joint and cognitive health, particularly in older adults [71]. Ubiquinol (coenzyme Q10), typically taken at 100–200 mg/day for adults, with elderly individuals benefiting from 100–300 mg/day due to better absorption, enhances mitochondrial function and supports cardiovascular and neurological health [96]. Lastly, tocotrienols, part of the vitamin E family, are supplemented at doses of 50–200 mg/day and offer superior antioxidant protection, supporting skin, cardiovascular health, and neuroprotection, particularly in elderly populations [51].

## 4. Food Supplements

### 4.1. Omega-3 Fatty Acids and Mechanisms of Action

Omega-3 fatty acid supplements are essential because the human body cannot synthesize these polyunsaturated fats [62]. The most well-known omega-3 supplements include eicosapentaenoic acid (EPA) and docosahexaenoic acid (DHA), which are derived from fish oils, and alpha-linolenic acid (ALA), which is available in plant-based supplements [100]. These omega-3 supplements play a vital role in numerous physiological processes, including anti-inflammatory responses, brain function, and energy metabolism [63,96]. More specifically, omega-3s contribute to the production of inflammatory eicosanoids, such as prostaglandins and leukotrienes, which counterbalance the pro-inflammatory compounds derived from omega-6 fatty acids [62]. Moreover, omega-3s generate resolvins and protectins, which actively resolve inflammation and prevent chronic inflammatory states [101]. Chronic inflammation is associated with fatigue, making omega-3 supplementation particularly beneficial for reducing fatigue in conditions like rheumatoid arthritis and chronic fatigue syndrome [102]. Finally, omega-3 fatty acids, particularly DHA, are also crucial for brain function. DHA is a key component of the phospholipid bilayer of brain cell membranes, facilitating neurotransmitter function and signal transduction [102]. DHA enhances synaptic plasticity, which is critical for learning and memory, and it supports cognitive function throughout life [103]. EPA and DHA have been shown to reduce symptoms of depression by modulating serotonin and dopamine pathways, which are vital for mood regulation [103].

#### Clinical Evidence on Omega-3 Supplementation

Clinical studies have shown that omega-3 supplementation can effectively reduce fatigue [25]. For instance, a randomized controlled trial demonstrated that EPA and DHA supplementation significantly lowered fatigue levels in patients suffering from chronic fatigue syndrome, likely due to their anti-inflammatory and energy-enhancing properties [104]. Omega-3 fatty acids have also been studied in individuals with major depression, where they were found to alleviate fatigue symptoms by improving mood and cognitive function [25].

In terms of cardiovascular health, omega-3 fatty acids support energy levels indirectly by improving vascular function. Studies have demonstrated that EPA and DHA reduce blood pressure, decrease triglyceride levels, and improve endothelial function, leading to better blood flow and reduced oxidative stress in vascular tissues [104].

Furthermore, one of the emerging areas of research on omega-3 supplements is their effect on the gut–brain axis [105]. Recent studies suggest that omega-3s may modulate the composition of gut microbiota, which in turn influences brain function and energy regulation [62]. This modulation of gut microbiota by omega-3 supplements plays a role in enhancing cognitive function and reducing stress-induced fatigue [63]. Omega-3s have been shown to reduce gut inflammation and improve gut barrier function, which can positively impact brain function through the bidirectional communication between the gut and the brain [106]. This gut–brain axis modulation may explain the fatigue-reducing effects of omega-3s in individuals with irritable bowel syndrome or other gut-related conditions.

Additionally, omega-3 fatty acids may play a role in hormonal regulation, particularly in managing cortisol, the body’s primary stress hormone. Chronic stress leads to elevated cortisol levels, which can contribute to fatigue and energy imbalances [106]. Omega-3s have been shown to reduce cortisol secretion, thereby supporting better stress management and improving overall energy balance [106]. This hormonal regulation makes omega-3 supplements a valuable tool in combating stress-induced fatigue and promoting sustained energy throughout the day [107,108].

In general, whether derived from fish oils or plant sources, omega-3 fats offer a wide range of health benefits, particularly in reducing inflammation, supporting brain function, and enhancing mitochondrial energy production. The clinical evidence supporting their role in fatigue reduction, cardiovascular health, and hormonal balance further solidifies omega-3s as a critical component of a balanced, energy-boosting diet [109].

### 4.2. Coenzyme Q10

Coenzyme Q10 (CoQ10), also known as ubiquinone, is a fat-soluble compound that plays a vital role in mitochondrial bioenergetics [110]. It is an essential component of the electron transport chain (ETC) in mitochondria, where it facilitates ATP production through oxidative phosphorylation [110]. CoQ10 is synthesized endogenously, but its levels decline with age, leading to impaired mitochondrial function and decreased energy production [111]. Consequently, CoQ10 supplementation has gained attention for its potential to enhance energy metabolism and support cardiovascular health, particularly in aging populations and individuals with cardiovascular diseases [112]. Further, CoQ10 plays a critical role in mitochondrial bioenergetics by shuttling electrons between Complexes I and II to Complex III within the ETC. This electron transfer is a key step in ATP production, as it drives proton pumping across the mitochondrial membrane, creating a proton gradient necessary for ATP synthase activity [65]. By facilitating efficient electron transfer, CoQ10 ensures the optimal generation of ATP, the energy currency of cells [65]. When CoQ10 levels are low, electron leakage can occur, leading to increased production of reactive oxygen species (ROS) and oxidative damage, which impairs mitochondrial function and contributes to cellular aging [65].

Supplementation with CoQ10 has been shown to improve mitochondrial function and ATP production in conditions characterized by mitochondrial dysfunction, such as chronic fatigue syndrome and fibromyalgia [113]. Studies have demonstrated that CoQ10 supplementation can enhance physical performance by increasing ATP synthesis and reducing muscle fatigue in both healthy individuals and those with mitochondrial disorders [110]. These findings underline the critical role of CoQ10 in maintaining energy homeostasis and reducing fatigue related to mitochondrial dysfunction [110]. Further, beyond its role in mitochondrial bioenergetics, CoQ10 acts as a potent antioxidant, protecting cellular membranes and lipoproteins from oxidative damage. CoQ10 exists in both oxidized (ubiquinone) and reduced (ubiquinol) forms, with ubiquinol serving as the active antioxidant species [65]. In its reduced form, CoQ10 directly searches for free radicals, preventing lipid peroxidation in cellular membranes and preserving the integrity of mitochondrial and other organelle membranes [113]. This dual role as an electron carrier and antioxidant makes CoQ10 indispensable in protecting tissues, particularly in organs with high metabolic demands, such as the heart and skeletal muscles [114].

CoQ10’s role in cardiovascular health is also primarily related to its antioxidant properties and its function in energy production. The heart is one of the most metabolically active organs, relying heavily on ATP for continuous contraction and relaxation [113]. CoQ10 protects cardiovascular tissues by neutralizing free radicals and reducing oxidative stress, which is a key contributor to the development of cardiovascular diseases such as atherosclerosis, heart failure, and hypertension [114]. Clinical evidence supports the use of CoQ10 in managing heart failure. A landmark study, the Q-SYMBIO trial, demonstrated that CoQ10 supplementation significantly improved symptoms, reduced hospitalizations, and decreased mortality in patients with chronic heart failure [113]. These cardioprotective effects are attributed to CoQ10’s ability to improve mitochondrial function, enhance ATP production, and reduce oxidative stress in cardiac tissues [114].

Finally, a meta-analysis of randomized controlled trials found that CoQ10 supplementation significantly reduced systolic blood pressure in patients with hypertension [112]. Another study showed that CoQ10 improved endothelial function and decreased oxidative stress markers in patients with type 2 diabetes, further supporting its role in vascular health [114]. Additionally, CoQ10 supplementation has been associated with improved exercise tolerance and reduced angina symptoms in patients with coronary artery disease [112]. So, as research continues to explore the multifaceted roles of CoQ10, its use in both preventive and therapeutic interventions is likely to expand.

### 4.3. Adaptogens

Adaptogens are a unique class of herbs and natural substances that help the body resist various forms of physical, chemical, and biological stress [115]. They work by regulating the body’s stress response and enhancing resilience to prolonged or intense stress, which in turn can help maintain energy levels and improve overall well-being [116].

Adaptogens like ginseng and ashwagandha work primarily by influencing the HPA axis, the central stress response system [117]. The HPA axis is responsible for regulating the release of cortisol, the primary stress hormone. Adaptogens help normalize cortisol levels, preventing both excessive cortisol release in response to acute stress and reducing cortisol overproduction in chronic stress [118]. For example, studies have shown that ashwagandha can significantly lower cortisol levels, thereby reducing stress and improving energy levels [119]. In addition to regulating cortisol, adaptogens also modulate other key neurohormones involved in the stress response, including adrenaline and noradrenaline [120]. Ginseng, for instance, has been shown to reduce the overactivation of the sympathetic nervous system, which helps lower stress-induced physiological responses such as increased heart rate and blood pressure [118]. This stabilization of the autonomic nervous system contributes to better stress management and enhanced energy availability, allowing the body to perform better under stress [119].

Adaptogens also play a role in supporting immune function, particularly during periods of stress, when the immune system is often compromised [119]. Ginseng has been shown to enhance immune responses by increasing the production of natural killer cells and T lymphocytes, which help the body fight infections [118]. By supporting the immune system, adaptogens help reduce the risk of illness during times of stress, which indirectly helps maintain energy levels and overall health [117]. Furthermore, adaptogens may influence hormonal balance, which is another key factor in managing stress and maintaining energy [118]. Ashwagandha, for example, has been shown to have a regulatory effect on the thyroid and adrenal glands, which play a crucial role in energy metabolism and stress management [121]. Research has demonstrated that ashwagandha supplementation can normalize thyroid hormone levels in individuals with hypothyroidism, further supporting its role in maintaining energy and reducing fatigue [63,121].

Several clinical trials support the use of adaptogens for improving energy levels and stress management [122]. A double-blind, placebo-controlled study demonstrated that ashwagandha supplementation significantly reduced stress and anxiety levels in adults while also improving their overall energy and vitality [117]. Similarly, a meta-analysis of studies on ginseng concluded that it improves physical performance and reduces mental fatigue, particularly in individuals exposed to stress [119].

Therefore, adaptogens such as ginseng and ashwagandha offer a natural means of modulating the stress response and improving energy levels [118]. By influencing the HPA axis, regulating cortisol and other stress-related hormones, and enhancing mitochondrial function, adaptogens help the body resist stress and reduce fatigue. The growing body of clinical evidence supports their efficacy in improving physical and cognitive performance, reducing stress-related fatigue, and enhancing overall vitality [122].

### 4.4. Amino Acids and Protein Supplements

Amino acids are the building blocks of proteins and play a vital role in numerous physiological processes, including muscle growth, immune function, and energy metabolism [123]. Protein supplements, often derived from whey, casein, soy, or plant-based sources, provide a concentrated form of amino acids to support muscle repair, recovery, and overall health [96]. Amino acids are classified as either essential, meaning they must be obtained through supplementation, or non-essential, meaning the body can synthesize them [63]. Amino acid and protein supplementation are commonly used to enhance physical performance, promote muscle hypertrophy, and improve recovery from exercise-induced muscle damage [51].

Essential amino acids (EAAs) are particularly important for stimulating muscle protein synthesis (MPS) [124]. Among these, leucine plays a critical role in activating the mTOR pathway, which regulates protein synthesis and promotes muscle growth [125]. Leucine supplementation, often in combination with other branched-chain amino acids (BCAAs), has been shown to enhance MPS and reduce muscle protein breakdown, making it an effective supplement for athletes and individuals engaging in resistance training [126]. Studies have consistently demonstrated that consuming EAAs, particularly in the form of whey protein, significantly enhances MPS after resistance exercise, supporting muscle recovery and growth [127].

In addition, BCAAs (leucine, isoleucine, and valine) are a subset of essential amino acids that are metabolized primarily in muscle tissue rather than the liver, making them particularly important for muscle recovery and energy production during exercise [128]. BCAAs can serve as a direct source of energy during prolonged physical activity by promoting fatty acid oxidation and reducing muscle protein breakdown [128]. Research has shown that BCAA supplementation can decrease muscle soreness and improve recovery following intense exercise [124]. Furthermore, BCAAs may reduce exercise-induced fatigue by competing with tryptophan, an amino acid that increases serotonin production and contributes to central fatigue [123].

Furthermore, protein supplements are widely used to enhance muscle recovery and promote hypertrophy, particularly in individuals engaged in resistance training or high-intensity exercise. Whey protein, derived from milk, is one of the most used protein supplements due to its high bioavailability and rapid absorption [124]. Whey protein is rich in BCAAs, particularly leucine, which makes it highly effective at stimulating MPS and enhancing muscle recovery [124]. In comparison, casein protein, another milk-derived protein, is digested more slowly, providing a sustained release of amino acids and making it ideal for nighttime consumption to prevent muscle protein breakdown during sleep [123].

As plant-based diets have gained popularity, so have plant-based protein supplements, typically made from sources such as peas, rice, hemp, or soy [124]. These supplements offer a concentrated source of protein for muscle repair and recovery. While plant-based proteins generally have lower concentrations of essential amino acids compared to animal-based proteins, they can still effectively support muscle recovery and growth when consumed in sufficient quantities [123]. Combining different plant-based protein sources, such as rice and pea protein, can improve the amino acid profile and enhance the supplement’s overall effectiveness [124]. Soy protein supplements in particular have been shown to promote gains in muscle mass and strength similar to those seen with whey protein when consumed in appropriate amounts [125]. Additionally, plant-based protein supplements are a valuable option for healthcare professionals who may prefer plant-based sources to support overall health, muscle recovery, and energy metabolism during demanding work schedules [125].

Overall, amino acids and protein supplements are essential tools for supporting muscle growth, recovery, and overall health. The growing body of research highlights the importance of amino acid supplementation in enhancing physical performance, promoting muscle recovery, and supporting long-term health.

### 4.5. Micronutrients

Micronutrients, including magnesium, iron, and B vitamins, play essential roles in maintaining overall health and supporting critical biological functions [129,130]. These nutrients are crucial in metabolic pathways, energy production, red blood cell (RBC) formation, and nervous system function. Deficiencies in these micronutrients can lead to impaired metabolic processes, fatigue, cognitive decline, and other health issues [131].

More specifically, magnesium is involved in over 300 enzymatic reactions in the body, many of which are related to energy production and metabolism [132]. Magnesium plays a crucial role in adenosine triphosphate (ATP) synthesis, acting as a cofactor for ATP-dependent enzymes, and it is essential for the activation of ATP, the primary energy currency of cells [133]. It also supports glycolysis, the citric acid cycle, and oxidative phosphorylation, which are vital for energy production in the mitochondria [134]. In addition to its role in metabolism, magnesium is vital for maintaining healthy nervous system function. It regulates neurotransmitter release, neuronal excitability, and synaptic plasticity, all of which are important for memory and cognitive function [135]. Magnesium also plays a role in reducing oxidative stress and inflammation in the brain, protecting neurons from damage [132]. Studies have shown that magnesium deficiency is associated with an increased risk of neurological disorders such as depression, anxiety, and cognitive decline [134,136].

Additionally, iron is an essential micronutrient that plays a pivotal role in oxygen transport and energy metabolism. As a component of hemoglobin, iron binds to oxygen in the lungs and delivers it to tissues throughout the body, ensuring that cells have the oxygen needed for aerobic energy production [131].

In addition, vitamin B, including B1 (thiamine), B2 (riboflavin), B3 (niacin), B6 (pyridoxine), B9 (folate), and B12 (cobalamin), are vital for energy production, RBC formation, and nervous system function. These water-soluble vitamins act as coenzymes in numerous metabolic pathways, facilitating the conversion of macronutrients (carbohydrates, fats, and proteins) into usable energy [137]. More specifically, thiamine (B1) plays a critical role in the metabolism of carbohydrates and amino acids, particularly in the decarboxylation of pyruvate to acetyl-CoA, which is necessary for entry into the citric acid cycle [138]. Thiamine deficiency leads to impaired glucose metabolism, resulting in fatigue, weakness, and neurological disorders such as beriberi and Wernicke–Korsakoff syndrome [139]. Riboflavin (B2) is essential for the functioning of flavoproteins involved in oxidative phosphorylation and the electron transport chain, which are critical for ATP production [140]. Riboflavin deficiency can lead to reduced energy production and symptoms such as fatigue, sore throat, and anemia [129]. Niacin (B3) is involved in the synthesis of NAD and NADP, coenzymes that play essential roles in redox reactions and ATP production [141]. Niacin deficiency, known as pellagra, is characterized by fatigue, diarrhea, dermatitis, and cognitive impairment [130]. Pyridoxine (B6) is necessary for amino acid metabolism and neurotransmitter synthesis, including the production of serotonin, dopamine, and gamma-aminobutyric acid (GABA) [142]. B6 also supports immune function by promoting the production of lymphocytes and cytokines [130]. Deficiency in B6 can lead to neurological symptoms such as depression, confusion, and irritability [130]. Deficiencies in folate or B12 can result in megaloblastic anemia, characterized by the production of large, immature RBCs, leading to fatigue and weakness [143]. Additionally, B12 deficiency is associated with neurological symptoms, including numbness, tingling, and cognitive decline [143].

### 4.6. Functional Foods and Subproducts

Functional foods and subproducts represent an emerging area in nutrition, offering health benefits beyond basic nutrition [144]. These foods and products are specially designed to improve health, enhance energy, and support cognitive function by incorporating bioactive compounds that play a critical role in preventing disease and improving overall well-being [145].

Functional foods are a category of foods that offer health benefits beyond their basic nutritional value, containing specific bioactive compounds that can promote health and help prevent disease [146]. These foods have become increasingly valuable in modern nutrition due to their ability to address specific health concerns, such as managing oxidative stress, supporting energy metabolism, and enhancing cognitive function. The concept of functional foods emerged in Japan in the 1980s and has since gained global recognition as an essential part of health-promoting diets [146]. Their popularity derives from their potential to offer targeted health benefits such as antioxidant effects, anti-inflammatory properties, and immune support.

The health benefits of functional foods are attributed to the presence of bioactive compounds, natural substances found in various plants and animal sources [147]. These compounds offer a range of health-promoting properties, making functional foods more than just a source of basic nutrients [147]. Flavonoids, for example, are potent antioxidants found in fruits, vegetables, tea, and wine. They help neutralize free radicals in the body, reducing oxidative stress, which is a key factor in aging and the development of chronic diseases [148]. Quercetin, a well-known flavonoid found in apples and onions, has been shown to possess strong anti-inflammatory effects and is associated with a reduced risk of cardiovascular disease and improved cognitive health [148].

Several functional foods are particularly effective in reducing oxidative stress and enhancing energy levels, offering an excellent addition to health-conscious diets. Green tea, for instance, is rich in catechins, especially epigallocatechin gallate (EGCG), which is known for its powerful antioxidant properties [149]. Green tea not only helps reduce oxidative damage to cells but also enhances metabolism and supports cognitive function, making it a valuable tool for both mental and physical energy [147]. Berries, such as blueberries and raspberries, are another excellent source of antioxidants. They contain high levels of flavonoids and anthocyanins, compounds that help protect the body against oxidative damage while also supporting brain function and cardiovascular health [150].

Fermented foods like yogurt, kefir, and kimchi provide probiotics, which contribute to gut health and overall energy levels. Probiotics help balance the gut microbiota, improving digestion and immune function while also positively influencing mood and energy by reducing stress and fatigue [151]. Additionally, nuts and seeds, such as walnuts, flaxseeds, and chia seeds, are rich in omega-3 fatty acids, fiber, and protein. These nutrients not only support cardiovascular health but also enhance energy metabolism, reduce inflammation, and improve cognitive function [152]. Including these functional foods in one’s diet can significantly contribute to overall health and well-being [153].

#### 4.6.1. Recent Innovations in Functional Subproducts

In recent years, there has been a rise in the development of functional subproducts, specially formulated foods, beverages, and supplements designed to provide specific health benefits [154]. Functional drinks, for instance, are becoming increasingly popular for their ability to enhance energy and focus [155]. These beverages are often enriched with antioxidants, vitamins, adaptogens such as ginseng and ashwagandha, and amino acids to boost cognitive performance and reduce stress [25]. Kombucha and green tea-based energy drinks are examples of functional beverages that provide a refreshing and healthy alternative to sugary energy drinks [156].

Functional bars are another innovation in the field, providing a convenient, nutrient-dense option for on-the-go nutrition [155]. These bars typically contain a mix of proteins, fibers, omega-3s, vitamins, and minerals, all designed to support sustained energy release and improve cognitive function throughout the day [156]. Similarly, functional powders have gained popularity as a versatile addition to smoothies or drinks. These powders often include superfoods such as spirulina and maca root as well as protein, probiotics, and adaptogens to enhance energy levels, support digestion, and promote overall health [157].

#### 4.6.2. Safety, Dosage, and Consumption Patterns of Functional Foods

While functional foods and subproducts offer a range of health benefits, it is essential to use them correctly to maximize their effects and avoid potential side effects [151]. Evidence-based guidelines recommend specific dosages for bioactive compounds like omega-3 fatty acids, probiotics, and flavonoids to ensure their safe and effective use. For example, the recommended daily intake of omega-3 fatty acids for general health is about 250–500 mg, although higher doses may be needed for therapeutic purposes such as reducing inflammation or lowering triglycerides [158]. Similarly, probiotics should be consumed regularly in appropriate amounts to maintain a healthy gut microbiota, with the specific strain and dosage depending on individual health needs [151].

Safety considerations are also crucial, as some functional foods or subproducts may interact with medications, cause allergic reactions, or be contraindicated in certain populations, such as pregnant women [159]. For instance, individuals with certain autoimmune conditions may need to avoid specific probiotic strains, while others might need to be cautious about excessive intake of omega-3 supplements due to blood-thinning effects [103]. To ensure optimal results, it is also important to follow best practices for consumption, including timing, proper integration into the diet, and combining functional foods with other nutrient-dense options [151]. A well-balanced diet, paired with strategic use of functional foods and subproducts, can provide substantial health benefits without compromising safety [154].

In Table 2, the basic functional foods and subproducts are mentioned.

Healthcare professionals face several challenges and barriers when trying to implement effective dietary interventions, including time constraints, knowledge gaps, and regulatory concerns. The demanding nature of healthcare work often leaves little time for meal preparation, leading to poor dietary choices that negatively impact energy levels and stress management [160]. Additionally, many healthcare professionals lack adequate knowledge of nutrition and the appropriate use of supplements and functional foods, further limiting their ability to maintain a balanced diet [60,160]. Regulatory concerns and the potential risks of overuse of certain supplements also pose significant barriers, as professionals may inadvertently misuse or over-rely on these products without fully understanding their effects [160].

To address the previously mentioned challenges, several solutions are proposed. Institutions should provide greater support, such as offering healthy food options in cafeterias and implementing educational programs that focus on the role of nutrition in managing stress and maintaining energy levels [154]. Also, access to reliable information on functional foods and supplements as well as professional guidance from dietitians or nutritionists can help healthcare professionals make informed choices [161]. Finally, emphasizing moderation in supplement use and focusing on foods the primary source of nutrients can also reduce the risks associated with overuse [162].

### 4.7. Supplement Safety

Consulting healthcare providers before starting any supplement regimen is the most critical step, as it ensures supplements do not interact with existing medications or health conditions. Without professional guidance, individuals risk facing serious consequences such as allergic reactions, toxicity, or reduced efficacy of prescribed drugs [163]. Choosing reputable brands and clean labels is equally important to avoid contamination, incorrect dosages, or harmful additives found in unregulated or poor-quality products [164]. This is essential for ensuring that supplements meet safety standards and deliver their intended benefits [165].

Additionally, understanding dosages and potential interactions is the key to preventing health complications. Improper dosing or combining supplements without knowledge of how they interact can lead to toxicity or exacerbate pre-existing conditions [166]. Lastly, educational programs that provide evidence-based information help combat misinformation and promote the responsible use of supplements [166], enabling healthcare professionals and the public to use them more safely and effectively.

Ιn Table 3, we summarize the essential tips for safe supplement use.

Overall, the risks associated with supplements depend on the type and dosage. Fat-soluble vitamins like A, D, and E pose toxicity risks, with an estimated 25% risk of accumulation in body tissues, leading to hypervitaminosis [167]. Iron supplements also carry a 10% risk for individuals with conditions like hemochromatosis, where excessive iron storage can damage organs [167]. Omega-3 fatty acids, while generally safe, have a 5% risk of increasing bleeding, especially in individuals on anticoagulants [165,166,167].

### 4.8. Supplement Interactions

Certain supplements can interact with medications, other supplements, or health conditions, potentially leading to adverse effects [168]. Understanding these interactions is crucial for ensuring safe use. For example, supplements such as St. John’s wort, calcium, iron, and omega-3 fatty acids are known to interact with several medications, often reducing their efficacy or leading to harmful side effects [168]. Similarly, calcium and iron can interfere with the absorption of thyroid medications and antibiotics such as tetracycline, potentially rendering these treatments less effective [168]. Omega-3 fatty acids, while beneficial for heart health, can also interact with blood thinners like warfarin, leading to an increased risk of bleeding [168]. These interactions underline the importance of healthcare provider consultations before combining supplements with prescription medications.

Supplements such as omega-3 fatty acids, ginkgo biloba, and vitamin E carry an increased risk of bleeding, especially when taken with blood thinners or NSAIDs like aspirin or ibuprofen [169]. It is critical to monitor signs of excessive bleeding, such as easy bruising or prolonged bleeding, and to consult a healthcare provider to adjust dosages or discontinue use if necessary [169]. Additionally, minerals like calcium, magnesium, and zinc can impact the absorption of medications, so a time gap between taking these supplements and medications in required to avoid diminished effectiveness [169].

Supplements can provide specific benefits based on age, as nutritional needs, health concerns, and metabolic processes change throughout life. In Table 4, we present a summary of potential interactions of supplements.

## 5. Integration of Dietary Interventions into Healthcare Professionals’ Lifestyles

### 5.1. Customized Dietary Plans Based on Stress Levels

Healthcare professionals experience varying levels of stress based on workload and personal circumstances, necessitating tailored dietary interventions. For those with low to moderate stress levels, the focus should be on maintaining a balanced diet that includes regular meals, moderate caffeine intake, and antioxidant-rich foods [161,170]. Basic supplementation with omega-3 fatty acids can help maintain cognitive function and cardiovascular health [171].

For those under high stress, it is important to emphasize food and supplements that support adrenal health. Adaptogens like ashwagandha and rhodiola have been shown to help the body resist stress and reduce cortisol levels [172]. In addition, supplements such as magnesium and B vitamins support energy production and nerve function, helping to manage stress more effectively [32]. Functional foods such as fermented foods improve gut health, which is closely linked to stress and mental health, while green tea provides cognitive support [173].

For those experiencing acute stress or burnout, dietary interventions should focus on easily digestible, nutrient-dense foods to prevent digestive overload [174]. Supplements like CoQ10, lipoic acid, and NAC can support mitochondrial health and reduce oxidative damage caused by chronic stress [110].

In Table 5, we report on a dietary plan for healthcare professionals.

According to the above table, healthcare professionals face varying stress levels and workloads, making it essential to tailor dietary strategies to meet their specific needs [110]. High-stress environments increase oxidative stress and demand more from the body’s energy systems, necessitating a diet that supports both cognitive and physical performance [175]. For individuals experiencing moderate stress, maintaining a balanced diet rich in fruits, vegetables, whole grains, lean proteins, and healthy fats are the key. These foods provide essential nutrients such as vitamins, minerals, and antioxidants that help reduce oxidative stress and maintain stable energy levels throughout the day [176].

For professionals facing high levels of stress, the inclusion of adaptogens such as ashwagandha and rhodiola can be beneficial in managing cortisol levels and reducing the physiological effects of stress [177]. Additionally, incorporating foods rich in omega-3 fatty acids, such as fatty fish and flaxseeds, can reduce inflammation and improve mental resilience [31]. For those experiencing chronic stress or burnout, it is crucial to prioritize nutrient-dense, easily digestible meals that do not burden the digestive system. In these cases, supplements like CoQ10 and lipoic acid can support mitochondrial function and help restore energy levels [111].

### 5.2. Optimizing Energy for Healthcare Professionals

Healthcare professionals often struggle with maintaining consistent energy levels due to irregular meal patterns and long shifts [173]. Meal ideas for healthcare workers should emphasize nutrient density and a balance of macronutrients to promote sustained energy. Breakfast options such as overnight oats with chia seeds, berries, and almonds offer a rich source of fiber, antioxidants, and healthy fats, helping to maintain blood sugar levels throughout the morning [156]. For lunch, a quinoa salad with leafy greens, grilled chicken, avocado, and a citrus dressing provides a well-balanced combination of protein, healthy fats, and antioxidants that support cognitive function and prevent energy crashes [178]. Also, dinner can include options like salmon with roasted vegetables and a side of brown rice, which provides omega-3 fatty acids, fiber, and complex carbohydrates to support recovery and sustained energy during the evening. Incorporating antioxidant-rich vegetables such as kale, spinach, and carrots further enhances the meal’s ability to reduce oxidative stress [179]. Meals should be easy to prepare, nutrient-dense, and tailored to individual preferences to ensure adherence. Snacks play a crucial role in maintaining energy levels during long shifts, providing a quick and convenient source of nutrients. Ideal snacks for healthcare professionals should combine protein, healthy fats, and fiber to sustain energy and prevent hunger [173]. Examples include Greek yogurt with berries, which offers probiotics for gut health along with antioxidants to combat oxidative stress [180,181]. Another easy option is raw nuts and seeds, such as almonds, walnuts, or sunflower seeds, which are rich in omega-3 fatty acids, magnesium, and protein, all of which support energy metabolism and reduce fatigue [149].

For healthcare professionals seeking more portable snacks, protein bars made with whole ingredients like oats, chia seeds, and natural peanut butter can provide a balance of carbohydrates and protein for sustained energy [178]. Including fruit and nut mixes with dried fruits like apricots or raisins offers a natural source of energy from sugars, while the fiber from the nuts helps stabilize blood sugar levels [182]. These snack options are not only convenient but also rich in nutrients that are essential for maintaining high energy levels during demanding work hours.

Supplements can play a significant role in helping healthcare professionals manage stress, enhance energy levels, and protect against oxidative damage. For general stress management, adaptogens such as ashwagandha have been shown to reduce cortisol levels and improve stress resilience [172]. Additionally, omega-3 fatty acids, especially EPA and DHA, can reduce inflammation and improve mental clarity, making them beneficial for healthcare workers under high stress [178].

For energy enhancement, coenzyme Q10 (CoQ10) is recommended due to its role in mitochondrial bioenergetics. CoQ10 improves energy production at the cellular level, helping reduce fatigue, especially in high-stress or physically demanding environments [120]. Another supplement, magnesium, supports muscle function and energy production and can also aid in relaxation and sleep quality, which is critical for recovery from stressful shifts [183].

Finally, healthcare professionals may benefit from B vitamins, particularly B6 and B12, which are involved in energy metabolism and the reduction of fatigue [130]. Tailored supplement recommendations should be guided by individual health profiles, stress levels, and dietary needs, ensuring that dosages are appropriate and effective for each professional’s lifestyle [154].

### 5.3. Institutional Support

Healthcare institutions must provide institutional support that encourages and facilitates healthy dietary choices [154]. One effective approach is offering healthy cafeteria options that prioritize antioxidant-rich, nutrient-dense foods. These should include a variety of fruits, vegetables, lean proteins, and whole grains as well as functional drinks like green tea and smoothies fortified with adaptogens [182]. Providing healthier options in vending machines and cafeterias ensures that healthcare professionals have easy access to nutritious food even during busy shifts [154].

Also, meal break policies should be enforced to guarantee that healthcare professionals have adequate time and space to eat without interruptions. In fast-paced healthcare environments, breaks are often skipped or cut short, leading to poor eating habits and increased stress [114]. Allowing time for regular, balanced meals will help staff maintain energy and reduce the risk of burnout.

Providing on-site nutrition services is another important step. Having nutritionists and dietitians available on-site allows healthcare professionals to receive expert advice tailored to their unique needs [167]. Additionally, employee wellness programs should incorporate dietary interventions, including meal planning workshops, cooking classes, and group nutrition challenges, to encourage participation and create a supportive culture around healthy eating [154]. These programs will not only improve the health of healthcare workers but also demonstrate a commitment to their well-being, which can improve job satisfaction and productivity (Table 6).

## 6. Case Studies and Practical Applications

Healthcare institutions have made significant strides in incorporating dietary interventions into wellness programs, leading to improved health outcomes. For example, the Mayo Clinic’s Integrative Medicine and Health Program combines nutrition, supplements, and mind–body practices for healthcare professionals. The program emphasizes antioxidants such as vitamins C and E, omega-3 fatty acids, and adaptogens like ashwagandha to manage stress and improve energy levels. The reported outcomes included a decrease in burnout and enhanced well-being among participants, particularly when supplements were combined with yoga and meditation [184]. Similarly, the Cleveland Clinic implemented a wellness program for staff that includes dietary interventions such as omega-3s, magnesium, and probiotics. The program provides access to dietitians and on-site healthy meal options based on personalized nutrition assessments, resulting in improved self-reported energy levels, reduced stress, and better gastrointestinal health [184]. Finally, a pilot program at St. Thomas’ Hospital in the U.K. focused on providing night-shift nurses with nutritional support, including vitamin D, B-complex vitamins, and omega-3 supplements. Blood tests identified specific deficiencies, which were addressed through tailored supplementation, improving mood, concentration, and immune function. The program also led to reduced absenteeism and enhanced energy levels among participants, demonstrating the importance of personalized supplementation plans for healthcare workers in demanding roles [184].

## 7. Clinical Trials and Intervention Studies

Clinical trials provide strong evidence of the effectiveness of dietary supplements in reducing stress and enhancing energy in healthcare professionals. A randomized controlled trial in the USA studied the impact of omega-3 fatty acids on stress reduction among nurses. Participants received 1500 mg of omega-3 fatty acids (EPA/DHA) daily for 12 weeks. The study showed a significant reduction in perceived stress and improved heart rate variability, indicating that omega-3 supplementation effectively reduces stress and improves cardiovascular health [184]. Also, a German cohort study involving 100 physicians assessed the effects of antioxidants (vitamins C and E, selenium, and glutathione) over six months. The results showed reductions in burnout scores (measured by the Maslach Burnout Inventory) and oxidative stress markers. These benefits were enhanced when antioxidant supplementation was paired with lifestyle changes such as yoga and cognitive–behavioral therapy [184]. Finally, another study in Canada examined the use of adaptogens such as *Rhodiola rosea* and ashwagandha to enhance cognitive function and reduce fatigue in healthcare professionals working night shifts. The double-blind, placebo-controlled trial found that participants who took adaptogen supplements for eight weeks showed significant improvements in cognitive function (measured by the Montreal Cognitive Assessment), reduced fatigue, and better sleep quality compared to the placebo [184]. These findings highlight the potential of dietary supplements to support both physical and mental health in high-stress professions.

## 8. Lessons Learned and Best Practices

Insights from the previously mentioned case studies and clinical trials reveal several best practices for implementing dietary interventions in healthcare settings. One of the most critical lessons is the importance of personalized nutrition plans, tailored to the specific needs of healthcare workers based on factors such as work schedules and stress levels. For instance, at St. Thomas’ Hospital, individualized supplementation plans for night-shift nurses helped address deficiencies, leading to improved health and reduced absenteeism. Another key factor is institutional support for healthy eating, which can be provided through on-site meal options, snacks, and access to dietitians. Cleveland Clinic’s wellness program, which included nutritionist consultations and healthy cafeteria options, resulted in greater adherence to dietary interventions and better health outcomes [184].

A combined approach to supplements and lifestyle modifications has also proven effective in reducing stress and burnout. The German study on antioxidant supplementation showed that pairing dietary interventions with stress-reduction techniques such as yoga and cognitive–behavioral therapy resulted in significantly lower burnout scores and oxidative stress [185]. Finally, ongoing education and awareness programs are essential for ensuring that healthcare professionals understand the benefits of proper nutrition, supplements, and functional foods in managing stress and enhancing energy [186].

Similarly, the Cleveland Clinic’s Wellness Program tailored personalized supplement plans for healthcare staff, focusing on omega-3s, magnesium, and probiotics. The intervention, which also included support from dietitians and access to healthy food options, resulted in improved energy levels, reduced stress, and better digestive health for participating staff. Personalized nutritional assessments were found to be key in addressing individual health needs, offering a pathway to more effective interventions [184].

The case studies and clinical trials reviewed here highlight that integrating dietary supplements with other lifestyle modifications, such as yoga, meditation, and exercise, leads to better health outcomes. For example, the German study on physicians supplemented with antioxidants (vitamins C and E, selenium, and glutathione) found that participants who paired supplementation with stress-reduction techniques like yoga and cognitive–behavioral therapy experienced a more significant reduction in burnout scores and oxidative stress markers compared to those who only received supplementation [185]. These findings suggest that a multifaceted approach, incorporating both nutritional and lifestyle interventions, is essential for maximizing the benefits of dietary strategies in high-stress professions [185].

Personalizing dietary interventions based on individual needs—such as stress levels, shift type, and nutritional deficiencies—has proven to yield better adherence and results [187]. For instance, the pilot program at St. Thomas’ Hospital in the U.K., which provided supplements like vitamin D, B-complex vitamins, and omega-3 fatty acids to nurses working night shifts, tailored the supplementation to address deficiencies identified through blood tests. The result was a notable improvement in mood, concentration, and energy levels as well as reduced absenteeism. Another example is the Cleveland Clinic’s wellness program, where personalized nutrition assessments allowed healthcare workers to receive tailored dietary interventions, leading to significant improvements in self-reported energy levels and reductions in stress [180]. These examples underscore the importance of customization to meet the specific needs of healthcare workers, ensuring that they can effectively manage the demands of their roles.

Providing education on the appropriate use of supplements as well as institutional support through access to healthy foods and expert guidance is essential for the success of dietary interventions. The Cleveland Clinic’s wellness program showed that offering dietitian support, on-site healthy food options, and regular educational workshops on nutrition and stress management resulted in greater adherence to dietary recommendations and better overall outcomes for staff [180]. Similarly, the Mayo Clinic’s integrative approach includes educational programs on the use of antioxidants, omega-3s, and adaptogens, helping healthcare professionals make informed decisions about their nutrition and supplement use. These examples highlight the importance of creating a supportive environment where healthcare workers have access to reliable information and resources, allowing them to implement and maintain healthier dietary practices. Additionally, consistent education ensures that healthcare professionals remain up to date on the best practices for using supplements to manage stress and improve well-being.

Overall, the reviewed case studies and clinical trials provide strong evidence that dietary interventions, when properly integrated into healthcare professionals’ routines, can significantly reduce stress, enhance energy, and improve overall well-being. A multifaceted approach combining supplements with other lifestyle interventions, tailored to the individual’s needs, and supported by educational programs and institutional initiatives proves to be the most effective strategy for promoting health in high-stress environments like healthcare [180].

## 9. Challenges and Future Research Directions

One of the primary challenges is the limited understanding of how antioxidants, functional foods, and dietary supplements can be effectively integrated into high-pressure professional environments such as healthcare [96]. While studies have demonstrated that these interventions can reduce oxidative stress and improve energy levels, their practical application in daily routines remains unclear [33,96]. Healthcare professionals face unique stressors that may influence the efficacy of dietary interventions, underscoring the need for more targeted research that explores these effects in specific work environments [16,43]. Another significant challenge is the lack of long-term studies examining the sustained benefits of dietary antioxidants and supplements. Current research often focuses on short-term interventions, leaving gaps in understanding how prolonged use impacts health outcomes, energy levels, and cognitive function. Comprehensive longitudinal studies are needed to assess the long-term effects, particularly in high-stress populations such as healthcare professionals [40]. Further, technological integration also presents both a challenge and an opportunity. Innovations like mobile apps and wearable devices have the potential to track oxidative stress and personalize dietary interventions, yet their full utility remains underexplored [96,188]. Research should focus on evaluating the effectiveness of these technologies in real-time monitoring and optimizing nutrition strategies to improve outcomes such as fatigue and cognitive function [40].

Another barrier is the heterogeneity of existing studies. Research on dietary antioxidants and supplements varies widely in terms of methodology, populations studied, and outcomes measured, making it difficult to draw consistent conclusions. Future studies should aim for standardization in design, interventions, and outcome measures to ensure more reliable and generalizable results [13,114]. Furthermore, the potential interactions between dietary supplements and medications commonly used by healthcare professionals must be explored. Given the high rates of medication use in this population, understanding how antioxidants and functional foods interact with pharmaceuticals is crucial to ensuring both safety and efficacy [51,114].

Additionally, an important issue for future research is improving the bioavailability of antioxidants from plant sources, which remains a critical area of study to maximize their health benefits. Bioavailability, defined as the proportion of a nutrient or bioactive compound that is absorbed and utilized by the body, is often limited in plant-derived antioxidants due to challenges such as poor solubility, instability during digestion, and extensive metabolism in the gastrointestinal tract [189]. One of the major challenges we face today concerning plant-derived antioxidants is their low solubility and stability, as many of them, such as polyphenols and carotenoids, are lipophilic or hydrophilic in nature, leading to poor absorption in digestive fluids [190]. During digestion, these antioxidants often undergo metabolic transformations, which may diminish their bioactivity. Moreover, the gut microbiota plays a role in breaking down polyphenols into bioactive metabolites, but individual variations in microbiome composition affect the efficiency of this process [191,192]. Additionally, the food matrix can influence antioxidant bioavailability, as complex plant structures can trap bioactives and limit their release during digestion [193]. Interactions with other dietary components, such as fibers binding to antioxidants, further hinder their absorption [194]. Future research should focus on developing advanced delivery systems to address these challenges [195,196]. As we know, techniques such as nano-encapsulation, liposomes, and emulsions can protect antioxidants during digestion and improve their absorption in the intestine [189,195]. Encapsulation methods also provide an opportunity to target specific release sites within the gastrointestinal tract, and the type and form of it could also be an important future research approach [197]. Finally, investigating synergistic nutrient combinations, such as the interaction of flavonoids with vitamin C or carotenoids with dietary fats, could optimize dietary formulations to enhance antioxidant uptake for cases such as obesity [198].

Moreover, research on probiotics and prebiotics that influence the microbial breakdown of polyphenols could offer new strategies to improve the bioavailability of these compounds [199]. Additionally, processing techniques such as fermentation, moderate heat treatments, and enzymatic hydrolysis can release antioxidants trapped within plant matrices without compromising their bioactivity [200]. Particle size reduction through micronization also increases the surface area of antioxidants, enhancing their solubility and absorption [201]. Overall, long-term bioactivity studies are essential to understanding how absorbed antioxidants are utilized in human tissues and their systemic effects on health [202]. While improving bioavailability is important, evaluating the functional impact of these compounds in the body will provide a comprehensive understanding of their benefits and forward research for alternative products derived for example from food waste and by-products [203].

Lastly, future research should focus on personalized nutrition approaches based on individual genetic, metabolic, and microbiome profiles that hold the potential for tailoring antioxidant-rich diets to maximize health outcomes [204,205]. Variability in individual responses to dietary interventions—due to factors like genetics, baseline nutritional status, and work environment—suggests that a one-size-fits-all approach may not be effective [206,207].

In conclusion, addressing the challenges of bioavailability requires a multifaceted approach that integrates advanced delivery systems, optimized processing methods, and personalized dietary strategies. These efforts, combined with a deeper understanding of antioxidant interactions with the gut and other dietary components, have the potential to unlock in the future the full health benefits of plant-derived antioxidants and contribute to the prevention and management of oxidative stress-related diseases for healthcare professionals and all people working in stressful environments.

## 10. Digital Tools, AI, and Interdisciplinary Collaboration in Personalized Nutrition and Stress Management for Healthcare Professionals

A key issue for healthcare workers is maintaining a routine with supplements due to long shifts and unpredictable schedules, which hinders the consistent intake that is essential for reducing oxidative stress [208]. Moreover, healthcare professionals often have knowledge gaps concerning the benefits of specific supplements. Despite their medical education, they may lack insight into the role of antioxidants in reducing stress-related conditions [16]. Providing evidence-based education on supplements can bridge these gaps [209]. Skepticism regarding supplements’ efficacy also poses a barrier for healthcare professionals [210]. Educational programs that deliver evidence-based guidance on the recommended duration and dosing of supplements can address this issue, as we learned with the experience of the COVID-19 period [211,212]. Studies suggest that at least 8 weeks of antioxidant use is necessary to see improvements in stress reduction and energy levels [213]. Also, combining supplements with holistic approaches such as CBT and yoga can offer additional benefits in reducing burnout [214].

Moreover, the integration of digital tools, apps, and AI in personalized nutrition offers an innovative avenue for real-time monitoring and tailored dietary recommendations [215]. Mobile apps that track nutrient intake, energy levels, and stress markers can help healthcare professionals stay on top of their dietary goals despite their busy schedules [216]. Also, AI-driven platforms could analyze individual data to recommend precise interventions, including supplement dosages and meal plans tailored to the user’s metabolic needs, stress levels, and work demands [217]. These tools could help overcome the barrier of time constraints and ensure that healthcare professionals receive personalized, science-backed dietary guidance.

Overall, for dietary interventions to be effectively implemented in healthcare settings, interdisciplinary collaboration is thus essential [218]. Nutritionists, psychologists, and healthcare administrators must work together to develop comprehensive wellness programs that address both the physical and psychological aspects of stress management in healthcare sectors [219,220]. Such programs should include not only dietary recommendations but also stress-reduction techniques like mindfulness training, yoga, and cognitive–behavioral therapy [218,219,220]. Integrating these approaches will ensure that healthcare professionals receive holistic care that promotes their overall well-being and enhances their capacity to provide high-quality care [221]. So, addressing challenges in implementing dietary and technological solutions for stress management in healthcare requires a multi-faceted approach. Time constraints, knowledge gaps, accessibility issues, and cultural barriers must be tackled [222,223]. Overcoming these obstacles will necessitate education, resource access, and organizational support to cultivate environments that support healthcare professionals’ well-being holistically and sustainably [224,225,226].

## 11. Conclusions

Healthcare professionals face high levels of oxidative stress due to the demanding nature of their work, contributing to fatigue and burnout. Antioxidant supplements such as vitamins C and E, omega-3 fatty acids, and CoQ10 have been shown to reduce oxidative damage and improve cognitive function and energy levels. Regular intake of these supplements, especially omega-3s, has been found beneficial. However, inconsistent supplementation due to long shifts and skepticism around efficacy presents challenges. Education on the benefits of antioxidants, combined with holistic approaches like cognitive–behavioral therapy (CBT) and yoga, can help reduce burnout. Yet, caution is needed, as excessive intake of fat-soluble vitamins may lead to toxicity. A personalized approach to supplementation that is supported by healthcare institutions is essential for the well-being and performance of healthcare professionals.

## Figures and Tables

**Table 1:** Antioxidants, food sources and Mediterranean diet tips for healthcare professionals.

Antioxidant	Natural Sources	Recommended Usage	Gender and Age Considerations	Specific Benefits	Diet Tips
Vitamin C (Ascorbic Acid)	Citrus fruits, strawberries, bell peppers, broccoli, kale, kiwi	Adults (18+): 75–90 mg/day; Smokers: Additional 35 mg/day;	Men and Women (18–50): General dosage for immune and antioxidant support; Pregnant Women: 85 mg/day; Elderly (50+): Supports immune function	Regenerates vitamin E, supports collagen synthesis, enhances immune function, and reduces oxidative markers data	Add fresh citrus fruits, bell peppers, and broccoli to salads; incorporate kiwi as a snack or dessert
Vitamin E (Tocopherol)	Nuts, seeds, spinach, sunflower oil, avocados	Adults (18+): 15 mg/day; Pregnant Women: 15 mg/day	Men and Women (18–50): Regular dosage for skin and cardiovascular health; Elderly (50+): Supports cognitive and cardiovascular health	Protects cell membranes from oxidation, improves skin and cardiovascular function, and reduces oxidative stress	Use extra virgin olive oil as the primary cooking oil; snack on a handful of nuts daily; add avocados to salads
Polyphenols	Tea (green and black), red wine, berries, apples, dark chocolate, nuts	Adults (18+): 200–1000 mg/day (as total polyphenol content); Higher Doses: Up to 1500 mg/day for therapeutic effects	Men and Women (18–50): Dosage varies by polyphenol type (e.g., quercetin, resveratrol); Pregnant Women: Consult a doctor	Modulates inflammatory pathways, protects DNA, supports vascular health, reduces inflammation, and improves cognitive function	Drink green tea or red wine in moderation; include berries and apples as daily snacks or dessert
Carotenoids (Beta-Carotene, Lycopene, Lutein)	Carrots, tomatoes, spinach, kale, sweet potatoes, cantaloupe, red peppers	Adults (18+): 6–15 mg/day (lutein); 2–4 mg/day (lycopene);	Men and Women (18–50): Supports eye, skin, and immune health; Pregnant Women: Ensure balanced intake; Elderly (50+): Supports cognitive health	Quenches singlet oxygen, protects lipids from peroxidation, supports eye health, improves immune function, and prevents skin damage	Add tomatoes and red peppers to Mediterranean-style salads; use spinach and kale in cooked dishes or smoothies
Astaxanthin	Microalgae, krill oil, salmon, shrimp	Adults (18+): 4–12 mg/day; Athletes: Up to 16 mg/day for enhanced performance	Men and Women (18–50): General dosage for skin and eye health; Elderly (50+): Protects against oxidative stress-related diseases	Improves skin elasticity, reduces oxidative stress markers, and supports muscle endurance	Include salmon or shrimp in weekly meals, such as grilled or in Mediterranean seafood dishes
Lipoic Acid (Alpha-Lipoic Acid)	Spinach, broccoli, tomatoes, organ meats (liver and heart)	Adults (18+): 300–600 mg/day; Diabetics: Up to 1200 mg/day under medical supervision	Men and Women (18–50): Antioxidant and energy metabolism support; Elderly (50+): Supports cognitive and glucose metabolism	Regenerates other antioxidants, enhances energy metabolism, and reduces oxidative stress; useful in managing diabetic neuropathy	Include sautéed spinach and broccoli as side dishes; organ meats like liver can be part of traditional Mediterranean cuisine
Glutathione (GSH)	Asparagus, avocados, spinach, garlic, unprocessed meat	Adults (18+): 250–500 mg/day (liposomal or S-acetyl glutathione); Therapeutic Use: Higher doses under medical supervision	Men and Women (18–50): General antioxidant and detoxification support; Elderly (50+): Supports immune function and energy levels	Master antioxidant for detoxification, immune function, and regulation of other antioxidants; may reduce fatigue	Add asparagus and garlic to Mediterranean-style vegetable dishes; use avocados in salads or spreads
N-Acetyl Cysteine (NAC)	Not found in foods directly; supplement form derived from cysteine, a component of high-protein foods (e.g., poultry and eggs)	Adults (18+): 600–1200 mg/day; Higher Doses (e.g., for lung health): Up to 2400 mg/day under supervision	Men and Women (18–50): For detoxification and antioxidant support; Elderly (50+): Supports cognitive and respiratory health	Precursor to glutathione, scavenges free radicals, replenishes intracellular GSH levels, and reduces oxidative damage in neurodegenerative disorders	Include high-protein foods such as poultry and eggs, which are staples in the Mediterranean diet, for supporting cysteine intake
Silymarin (from Milk Thistle)	Milk thistle seeds	Adults (18+): 200–400 mg/day (standardized to 70–80% silymarin); Liver Conditions: Up to 600 mg/day under medical supervision	Men and Women (18–50): For liver detoxification; Elderly (50+): Supports general liver health; Pregnant Women: Avoid due to safety concerns	Protects liver cells, supports detoxification, reduces lipid peroxidation, and enhances antioxidant enzyme activity	Milk thistle is not common in Mediterranean cuisine, but general liver health can be supported with other Mediterranean foods like olive oil and greens
Curcumin (from Turmeric)	Turmeric root	Adults (18+): 500–2000 mg/day with piperine for enhanced absorption; Higher Use: Up to 3000 mg/day for inflammatory conditions	Men and Women (18–50): General dosage for inflammation; Elderly (50+): Supports joint and cognitive health; Pregnant Women: Consult a doctor	Anti-inflammatory and antioxidant, modulates inflammatory pathways, and supports joint health; potentially beneficial for oxidative stress-related disorders	Add turmeric as a spice in Mediterranean dishes such as stews or marinades for an anti-inflammatory boost
Ubiquinol (Reduced Coenzyme Q10)	Small amounts in organ meats and fatty fish	Adults (18+): 100–200 mg/day; Elderly (50+): 100–300 mg/day due to better absorption rates	Men and Women (18–50): For cardiovascular and energy support; Elderly (50+): Enhanced absorption for energy and heart health	Enhances mitochondrial function, reduces fatigue, and supports cardiovascular and neurological health	Include fatty fish like sardines or salmon in Mediterranean dishes to naturally boost coenzyme Q10 intake
Tocotrienols (Vitamin E Family)	Palm oil, rice bran oil, annatto seeds	Adults (18+): 50–200 mg/day; Higher Needs (Anti-aging or Neuroprotective Use): Up to 300 mg/day	Men and Women (18–50): Regular antioxidant support; Elderly (50+): Neuroprotection and anti-aging benefits; Pregnant Women: General RDA applies	Superior antioxidant protection; supports skin health and cardiovascular health; neuroprotection	Incorpore extra virgin olive oil in place of palm or rice bran oil—a common practice in the Mediterranean diet

**Table 2:** Summary of functional foods and subproducts.

Functional Food/Subproduct	Bioactive Compounds	Natural Sources or Formats	Recommended Usage	Gender and Age Considerations	Specific Benefits
Green tea	Catechins (e.g., EGCG) and polyphenols	Green tea leaves and matcha powder	Adults (18+): 2–4 cups/day (up to 400 mg catechins); Pregnant Women: Limit to 2 cups/day due to caffeine content	Men and Women (18–50): General antioxidant and cognitive benefits; Elderly (50+): Supports cognitive function and metabolism	Reduces oxidative stress, enhances metabolism, supports brain function, and may aid in weight management
Berries (e.g., blueberries and raspberries)	Flavonoids (anthocyanins and quercetin) and vitamin C	Fresh, frozen, dried berries, berry powders	Adults (18+): 1–2 cups/day (fresh or frozen); Higher Needs: Up to 3 servings/day for enhanced antioxidant effects	Men and Women (18–50): Supports cardiovascular and cognitive health; Pregnant Women: Good source of nutrients, balanced intake	Protects against oxidative damage, supports brain and heart health, and enhances immune function
Fermented foods (e.g., yogurt, kefir, kimchi, sauerkraut)	Probiotics (Lactobacillus and Bifidobacterium), vitamins, minerals	Fermented dairy products, fermented vegetables, kombucha	Adults (18+): 1–2 servings/day; Higher Doses: For specific gut health conditions	Men and Women (18–50): Supports gut and immune health; Elderly (50+): Enhances digestion and nutrient absorption	Supports gut microbiota, enhances immune response, and may reduce stress-related fatigue
Nuts and seeds (e.g., walnuts, flaxseeds, chia seeds)	Omega-3 fatty acids, fiber, protein, vitamins (e.g., E and B)	Whole nuts and seeds, ground flaxseed, chia seed puddings, nut butters	Adults (18+): 1–2 oz/day (nuts); 1–2 tbsp/day (seeds); Pregnant Women: Focus on omega-3-rich nuts and seeds	Men and Women (18–50): For cardiovascular and brain health; Elderly (50+): Supports cognitive function and inflammation management	Reduces inflammation, supports heart and brain health, and enhances energy metabolism
Functional drinks (e.g., green tea energy drinks, kombucha)	Antioxidants (catechins and polyphenols), adaptogens, probiotics	Ready-to-drink bottles and powdered drink mixes	Adults (18+): 1–2 servings/day; Higher Needs (Athletes): Up to 3 servings/day based on activity level	Men and Women (18–50): For energy and focus; Elderly (50+): Cautious use due to caffeine; Pregnant Women: Avoid high caffeine	Supports energy, mental clarity, immune function, and gut health
Functional bars (e.g., protein bars, omega-3 bars)	Proteins, omega-3 fatty acids, fiber, vitamins (B and D), minerals (iron and magnesium)	Snack bars and meal replacement bars	Adults (18+): 1–2 bars/day as a snack or meal replacement; Athletes: Higher protein content bars post workout	Men and Women (18–50): For sustained energy; Elderly (50+): Focus on balanced nutrient content; Pregnant Women: Check for allergens	Provides sustained energy, supports muscle recovery, and is a convenient source of nutrients
Functional powders (e.g., superfood powders and protein powders)	Superfoods (spirulina and maca root), proteins, probiotics, adaptogens	Powdered supplements added to smoothies, shakes, or water	Adults (18+): 1–2 scoops/day depending on product; Higher Needs: For specific goals (e.g., muscle gain or gut health)	Men and Women (18–50): Supports energy and recovery; Elderly (50+): Focus on balanced nutrient content and digestibility	Enhances energy, supports recovery, and provides antioxidants, probiotics, and adaptogens

**Table 3:** Comprehensive tips for safe supplement use.

Safety Tip	Analysis	Potential Risks of Not Following
Consult with a healthcare colleague	Consulting a healthcare provider ensures personalized advice based on individual health status, medication use, and specific needs, reducing the risk of adverse effects.	Risk of harmful interactions with medications, exacerbation of pre-existing conditions, and inappropriate supplement choices.
Choose reputable brands	Reputable brands with third-party certifications (e.g., USP and NSF) adhere to quality standards, ensuring purity, potency, and safety of supplements.	Risk of contamination, substandard quality, inaccurate labeling, and ineffective or harmful ingredients.
Understand recommended dosages	Following recommended dosages prevents toxicity and ensures supplements are used safely and effectively, avoiding overuse or underuse.	Overdosing on certain vitamins (e.g., A, D, E, and K) can lead to toxicity; underdosing can lead to ineffective treatment and nutrient imbalances.
Be aware of potential interactions	Being aware of interactions with medications or other supplements prevents adverse effects and ensures safe concurrent use of treatments.	Severe interactions can occur, such as bleeding with fish oil and blood thinners or reduced effectiveness of medications (e.g., antibiotics).
Start with lower doses	Starting with a lower dose allows the body to adjust and helps identify any side effects or intolerances before taking the full dose.	There is potential for adverse reactions or intolerances (e.g., digestive issues and headaches) that could be severe with full doses.
Avoid “mega-dosing”	Avoiding high doses of supplements without medical supervision helps prevent toxicity, especially with fat-soluble vitamins (A, D, E, and K) and minerals (e.g., iron).	Risk of toxicity, such as liver damage (vitamin A), hypercalcemia (vitamin D), or hemorrhagic stroke (vitamin E).
Look for clean labels	Choosing supplements with minimal, recognizable ingredients reduces the risk of allergic reactions, interactions, and exposure to unnecessary additives.	Risk of allergic reactions, gastrointestinal distress, or negative health effects from artificial additives or allergens.
Monitor for side effects	Monitoring for side effects ensures timely identification of adverse reactions, allowing for discontinuation or adjustment of the supplement regimen.	Continued use despite side effects could lead to worsening health problems, such as gastrointestinal issues or allergic reactions.
Store supplements properly	Proper storage (cool, dry places; refrigeration for probiotics and fish oil) maintains the potency and safety of supplements, preventing degradation and contamination.	Degradation or contamination could lead to reduced efficacy or potential health risks from spoiled or contaminated products.
Read labels carefully	Reading labels carefully helps consumers understand dosages, ingredients, allergens, and any specific usage instructions or warnings.	Misuse of supplements due to misunderstanding label information can lead to overdosing, allergies, or ineffective use.
Rotate supplements	Rotating supplements, especially adaptogens or herbs, prevents tolerance build-up and reduces the risk of potential long-term negative effects.	Over-reliance or long-term use of certain supplements may lead to reduced efficacy, hormonal imbalances, or other health issues.
Stay informed with evidence-based information	Using credible sources ensures consumers are guided by reliable, research-backed information rather than marketing or anecdotal evidence.	Risk of falling for misleading claims, using ineffective products, or experiencing health risks from non-evidence-based practices.
Consider dietary sources first	Prioritizing nutrients from a balanced diet promotes overall health, reducing the need for high-dose supplements and their associated risks.	Relying on supplements without a balanced diet may lead to nutrient deficiencies, imbalances, and less optimal health outcomes.
Use caution with high-risk supplements	Caution with high-risk supplements (e.g., iron and fat-soluble vitamins) helps prevent overuse and ensures they are only taken when medically necessary.	Higher risk of toxicity, particularly in vulnerable populations (e.g., elderly and those with chronic conditions).
Report adverse reactions	Reporting adverse reactions ensures tracking of supplement safety, helps in regulatory oversight, and contributes to public health data.	Failure to report can lead to continued use of harmful supplements by others and a lack of regulatory action or safety updates.

**Table 4:** Common supplement interactions.

Supplement	Potential Interactions	Description of Interaction	Precautionary Measures
St. John’s Wort	Medications: Antidepressants (SSRIs and MAOIs), birth control pills, blood thinners (warfarin), immunosuppressants, anti-seizure drugs	Can decrease the effectiveness of medications (e.g., antidepressants and birth control) or increase the risk of serotonin syndrome and bleeding when combined with SSRIs.	Avoidance of St. John’s wort is recommended when taking certain medications, and consultation with a healthcare provider is essential before starting its use.
Vitamin K	Medications: Blood thinners (e.g., warfarin)	This can reduce the effectiveness of blood thinners by promoting clotting; this could lead to dangerous clotting conditions.	Consistent intake of vitamin K is advised, with monitoring of INR levels if on warfarin; consultation with a healthcare provider is recommended.
Calcium	Medications: Antibiotics (e.g., tetracyclines and quinolones) and thyroid medications (e.g., levothyroxine)	Calcium can bind to certain antibiotics or thyroid medications in the digestive tract, reducing their absorption and effectiveness.	Calcium supplementation should be timed differently from these medications, ensuring a 2–4-h separation.
Iron	Medications: Antibiotics (e.g., tetracyclines and fluoroquinolones), antacids, thyroid medications (levothyroxine)	Iron can decrease the absorption of certain medications and be affected by antacids, reducing its own absorption.	Separation of iron supplements from these medications by at least 2 h is advised, and high-dose iron supplementation should be avoided without medical guidance.
Omega-3 fatty acids	Medications: Blood thinners (e.g., warfarin and aspirin) and non-steroidal anti-inflammatory drugs (NSAIDs)	High doses of omega-3s can increase bleeding risk, especially when combined with blood thinners or NSAIDs.	Limitation of omega-3 supplement intake to 1000 mg/day is advised without medical consultation; discussion with a healthcare provider is recommended.
Magnesium	Medications: Antibiotics (e.g., tetracyclines and quinolones), diuretics, muscle relaxants, osteoporosis medications (bisphosphonates)	This can reduce the absorption and effectiveness of certain antibiotics and osteoporosis medications; it may potentiate the effects of muscle relaxants and diuretics.	Separation of magnesium supplementation from these medications by several hours is recommended, and verification of potential interactions with a pharmacist is advised.
Ginseng	Medications: Blood pressure medications, anticoagulants, stimulants, diabetes medications	This can affect blood pressure levels, increase the risk of bleeding, or interact with diabetes medications to affect blood sugar levels.	Avoidance of these medications unless supervised by a healthcare provider is recommended, with monitoring of blood pressure and glucose levels.
Vitamin D	Medications: Steroids (e.g., prednisone), weight-loss drugs (e.g., orlistat), cholesterol-lowering drugs (e.g., cholestyramine).	Some medications can reduce vitamin D absorption or increase its metabolism, reducing its effectiveness; high doses can lead to hypercalcemia.	Regular monitoring of vitamin D levels is recommended when on these medications, and avoidance of high-dose supplementation without a doctor’s advice is advised.
Ginkgo Biloba	Medications: Blood thinners (e.g., warfarin and aspirin), anticonvulsants, antidepressants, NSAIDs, diabetes medications	Increases the risk of bleeding when combined with blood thinners; can affect blood sugar levels or cause seizures with anticonvulsants.	Cautious use of these medications is recommended; consultation with a healthcare provider is essential, especially when on blood thinners.
Zinc	Medications: Antibiotics (e.g., tetracyclines and quinolones), diuretics, immune-suppressing drugs (e.g., cyclosporine)	Zinc can interfere with the absorption of certain antibiotics and increase the risk of side effects with immune-suppressing drugs.	Zinc supplementation should be taken separately from these medications, and guidance from a healthcare provider is advised
Melatonin	Medications: Sedatives (e.g., benzodiazepines), blood pressure medications, anticoagulants, immunosuppressants, contraceptives	It can potentiate the effects of sedatives, affect blood pressure levels, increase bleeding risk, or interfere with the efficacy of immunosuppressants and contraceptives.	Cautious use of these medications is advised, with a discussion about potential interactions with a healthcare provider.
Coenzyme Q10 (CoQ10)	Medications: Blood pressure medications, chemotherapy drugs, blood thinners (e.g., warfarin).	May reduce the effectiveness of blood thinners, lower blood pressure excessively, or interfere with chemotherapy drugs’ effectiveness.	Monitoring blood pressure and INR levels is essential, and consultation with a healthcare provider is recommended before using CoQ10 when on these medications.

**Table 5:** Proposed dietary plan for healthcare professionals based on stress levels.

Stress Level	Dietary Strategy	Breakfast Ideas	Lunch Ideas	Dinner Ideas	Snack Options	Supplement Recommendations
Low to moderate stress	Focus on a balanced diet with antioxidant-rich foods, regular hydration, and moderate caffeine intake.	Oatmeal with berries, chia seeds, and a drizzle of honey; green smoothie with spinach, banana, and flaxseed.	Grilled chicken salad with mixed greens, avocado, nuts, and a vinaigrette; quinoa and vegetable stir-fry with tofu.	Baked salmon with steamed broccoli and brown rice; lentil soup with a side of whole-grain bread.	Apple slices with almond butter; Greek yogurt with honey and walnuts; a handful of mixed nuts and dried fruits.	Omega-3 Fatty Acids: 1000 mg/day; Multivitamin: One daily with balanced micronutrients; Probiotics: One serving/day of fermented foods or supplement.
High stress	Increase intake of adaptogens, magnesium, B vitamins, and antioxidant-rich foods to support adrenal and cognitive function.	Whole-grain toast with avocado, poached eggs, and pumpkin seeds; smoothie bowl with spinach, berries, and protein powder.	Turkey and avocado wrap with leafy greens and hummus; chickpea and quinoa salad with mixed vegetables, olives, and feta cheese.	Grilled chicken or tofu with sweet potato, green beans, and tahini sauce; stir-fried shrimp with vegetables and brown rice.	Dark chocolate with nuts; carrot sticks with hummus; green tea with lemon and honey; cottage cheese with fresh berries.	Ashwagandha or Rhodiola: 300–600 mg/day; Magnesium Glycinate: 200–400 mg/day; B-Complex Vitamins: One tablet/day with meals for energy support.

**Table 6:** Challenges, barriers, and solutions for implementing dietary interventions among healthcare professionals.

Challenge/Barrier	Description	Impact	Proposed Solutions
Time constraints and accessibility	Limited time for meal preparation, irregular eating patterns, and lack of access to healthy options.	Leads to reliance on fast food or snacks, irregular nutrient intake, decreased energy levels, and difficulty in consistent supplement use.	Institutional support by providing healthy meal options in cafeterias; meal planning by offering quick, easy-to-prepare recipes; provision of ready-to-go supplement packs for healthcare workers.
Knowledge gaps and misconceptions	Misunderstandings about the benefits, dosages, and safety of antioxidants, supplements, and functional foods.	Results in improper use, over-reliance on supplements, and potential health risks due to misinformation or inappropriate usage.	Educational programs such as workshops on nutrition and supplements; access to reliable resources so as to provide evidence-based guidelines and materials; consultation services with access to dietitians and nutritionists.
Regulatory and safety concerns	Variability in regulation and quality of supplements; potential interactions with medications and safety concerns with long-term use.	Increases risk of consuming low-quality products, adverse interactions with medications, and health complications due to unregulated use.	Quality assurance with reccomendation of reputable brands and certified products; professional guidance to encourage regular consultations with healthcare providers; policy development with clear guidelines on supplement use.
Addressing potential risks and overuse	Risks of toxicity and negative health effects from overconsumption of certain antioxidants and supplements; need for moderation and evidence-based practices.	Potential for serious health complications such as liver damage, hypercalcemia, and pro-oxidant effects, leading to increased health risks rather than benefits.	Moderation emphasis that promotes the importance of balanced use and recommended dosages; monitoring programs with regular check-ups to assess supplement use; research-based guidelines with updated protocols for safe use.
